# College of Radiology, Academy of Medicine of Malaysia position on whole body screening CT scans in healthy asymptomatic individuals (2008)

**DOI:** 10.2349/biij.4.4.e44

**Published:** 2008-10-01

**Authors:** ELM Ho, BJJ Abdullah, AAL Tang, AJ Nordin, AR Nair, GCC Lim, H Samad-Cheung, KH Ng, S Ponnusamy, SF Abbas, SI Bux, S Arasaratnam, YF Abdul Aziz, S Venugopal, Z Musa, Z Abdul Manaf

**Affiliations:** 1 College of Radiology, Academy of Medicine of Malaysia; Sime Darby Specialist Centre Megah, Petaling Jaya, Malaysia; 2 University Malaya Medical Centre, Kuala Lumpur, Malaysia; 3 Sime Darby Medical Centre Subang, Selangor, Malaysia; 4 Universiti Putra Malaysia, Selangor, Malaysia; 5 Pantai Medical Centre, Kuala Lumpur, Malaysia; 6 Kuala Lumpur Hospital, Kuala Lumpur, Malaysia; 7 Islamic International University of Malaysia, Pahang, Malaysia; 8 Sungai Buloh Hospital, Selangor, Malaysia; 9 Malacca Hopital, Malacca, Malaysia; 10 Hospital Tuanku Ja’afar Seremban, Negeri Sembilan, Malaysia; 11 Selayang Hospital, Malaysia

**Keywords:** Screening, computed tomography (CT), radiation risks, cost-benefit analysis, whole body imaging

## Abstract

To date, the College of Radiology (CoR) does not see any clear benefit in performing whole body screening computed tomography (CT) examinations in healthy asymptomatic individuals. There are radiation risk issues in CT and principles of screening should be adhered to. There may be a role for targeted cardiac screening CT that derives calcium score, especially for asymptomatic medium-risk individuals and CT colonography when used as part of a strategic programme for colorectal cancer screening in those 50 years and older. However, population based screening CT examinations may become appropriate when evidence emerges regarding a clear benefit for the patient outweighing the associated radiation risks.

## PREAMBLE

After examining the published literature to date, the College of Radiology (CoR) does not see any clear benefit in performing whole body screening computed tomography (CT) examinations in healthy asymptomatic individuals. This recommendation is made based on available evidence and the points taken into consideration are discussed in the various sections below. It does not replace case-by-case or individual clinical assessment where the need arises. It is also not meant to be used for legal purposes.

This position provides guidance for medical and health care professionals for appropriate radiologic care that is as effective and safe as possible for the patient. It is recognised that many factors come into play in the delivery of health care. This includes the patient's condition, available resources and new information, results from studies, as well as new technologies.

All parties, i.e. the radiologist who agrees to perform and interpret this examination, the physician prescribing the examination (if the patient is not self-referred) as well as the patient, must fully understand the limitations and implications of the findings, as well as the risks entailed (from the radiation and contrast media). All must be cognisant of the fact that there may be ensuing investigations for abnormalities found on the CT scan and these may have potentially profound financial, psychological and physical effects.

## INTRODUCTION

Sectional imaging such as Computed Tomography (CT) or Magnetic Resonance Imaging (MRI) have revolutionised the capabilities for medical imaging studies, image-guided therapeutic intervention, and improved targeted radiation therapy. Unlike non-ionising ultrasound examinations, it is not limited by gas and body habitus or conventional radiographic imaging where there is overlapping of significant volumes of tissue. CT provides visualisation of body structures previously not well visualised except through open surgery. CT is much more widely available and therefore much more utilised than MRI. CT has benefited many patients by clinching the diagnosis, guiding surgery, staging a disease much more accurately and allowing radiation therapy to proceed ever more precisely.

The varieties of CT scanners currently in clinical use range from single slice to spiral, multislice and dual source technology as well as electron beam CT (EBCT). Scans can now proceed more rapidly by acquiring each sectional image of the body in subsecond or millisecond acquisition times. Multislice, dual source and EBCT allow elegant multiplanar and 3-D reconstructions as well. Virtual colonoscopy is one good example where post processing 3-D rendering has been invaluable.

Therefore, it is not surprising that CT is now being explored and employed in whole body screening for disease. In fact, screening CT centres have sprouted up based on the premise of wellness screening – to detect diseases before they become more advanced. A whole body CT is being marketed directly to consumers (patients), and they, in turn, are now demanding for the test.

## DOES WHOLE BODY SCREENING CT SATISFY THE CRITERIA OF A GOOD SCREENING TEST?

The following are intended as points of consideration rather than questions. All focus on whether whole body screening CT meets the standards of a good screening test for the target diseases (diseases generally being marketed for are cancer and coronary artery disease) [1,21^3^]. Do note that whole body screening CT may include the head, neck, thorax, abdomen and pelvis. In some centres or countries, this may only include the neck, thorax, abdomen and pelvis, or just the thorax, abdomen and pelvis.

1. Results from large-scale randomised clinical trials (RCT) for evaluation of whole body screening in apparently healthy individuals has not been published to date. These studies may be difficult to perform because of ethical issues regarding radiation dose, being very significant in whole body CT as well as the costs of the examination and the risks from contrast media, if administered in routine whole body screening CT. In addition, RCTs generally tend to be disease-specific, and interpreting results of trials where an individual is being screened for multiple diseases at the same time may not be straightforward. In addition, CT technology is rapidly advancing and therefore by the time an RCT is concluded, would the results be applicable?
2. Indices for whole body CT such as negative predictive rate, sensitivity, specificity, false negative, false positive and others are unknown. Therefore the cost-effectiveness or more comprehensively, the benefit-risk-cost evaluation is lacking.
3. Malpractice issues have to be considered in screening CT such as would arise if there was a missed diagnosis and/or where intravenous contrast media used resulted in a severe contrast reaction. Linked to the possibility of missed diagnosis, is the question of whether intravenous contrast media should be routinely used for whole body screening CT examinations. It is known that non-enhanced CT of the abdomen is generally inadequate for lesion characterisation. [4]
4. No specific guidelines exist for follow-up or further evaluation when abnormalities are detected for general whole body screening. Currently there are some guidelines or consensus statements for lung CT but this is because there is a basis for the size or other pretest probabilities. Guidelines for lung CT (relatively organ specific) are easier to establish than for abdomen CT (multiple organs).

## PRINCIPLES IN SCREENING

Screening involves a test, procedure or investigation that is used to identify a condition of disease before it manifests with signs and symptoms. It is based on the premise that detecting a disease at its earliest affords the best chance of cure.

A screening test is considered effective if it produces a statistically significant reduction in disease mortality. Screening tests can be applied to the whole population or to a subset of the population, for example, that is based on the risk profile of the person for a specific disease.

Main considerations for screening are as follows [3]:

1. Does the disease merit screening? (For example, is the disease very common in the population in question and is proving to be a major healthcare burden to treat and care for?)
2. Is there a reliable screening test for the disease in question? The false positive and false negative rates, positive and negative predictive values as well as accuracy are important parameters.
3. Is effective intervention/treatment available for the disease in question, if detected early? There is no point in screening for a disease if there is no cure or effective treatment for the disease. There is also no point in treating a disease that is slowly progressing such that treating it early may not make a difference to the patient’s lifespan.
4. Therefore, screening in general requires the careful consideration of a number of factors including a careful cost-benefit analysis. Individuals opting for any screening examination should be counseled as to the benefits and risks associated with the examination.

## RADIATION ISSUES IN CT

The effective doses from diagnostic CT procedures are typically estimated to be in the range of 1 to 10 mSv. However, for a whole body CT, the effective dose for CT head, thorax, abdomen and pelvis is easily 20 mSv [5]. A CT examination with an effective dose of 10 mSv may be associated with an increase in the chance of fatal cancer of approximately 1 in 2000. Therefore, a CT scan must only be performed when the benefits outweigh the risks, and when information from the CT scan will positively affect the management of the patient’s condition.

The amount of radiation dose received by the patient is variable by a factor of 10 [6, 7], depending on the size of the patient, make and type of CT scanner, scanning parameters and body part being scanned. It is also an established fact that CT studies account for the largest population radiation dose from medical diagnostic studies and this is increasing rapidly with time [8,9,10]. In 1998, CT contributed 42% of the average effective dose per capita from diagnostic exposures in the Netherlands [8].

According to the UNSCEAR 2000 Report on Sources and Effects of Ionising Radiation, the percentage contribution by CT scans to global collective dose from medical x-ray examinations have increased from about 14% between 1955 and 1990 to about 33% between 1991 and 1996 [9]. The growth is phenomenal and, therefore, the collective dose to the population from medical sources of ionising radiation is significant.

## THE COLLEGE OF RADIOLOGY, ACADEMY OF MEDICINE POSITION ON WHOLE BODY SCREENING CT SCANS

After examining the published literature to date, the College of Radiology (CoR) does not see any clear benefit in performing whole body screening computed tomography (CT) examinations in healthy asymptomatic individuals. Indiscriminate whole body screening CT does not satisfy the criteria needed for a good screening test. There is also no standardisation of what constitutes a whole body CT scan and whether contrast media should be used. There is no evidence-based consensus of the various diseases for which morbidity and mortality are allegedly reduced. It should be noted that there is no “one size fits all” for a properly conducted CT scan, especially so if the whole body is involved. In addition, a CT scan examination is costly when it involves many sections of the body (as in whole body) and contrast media is used with attendant risks.

The false positives from the whole body screening CT will lead to additional tests, some of which may be high risk (for example fatality from biopsy procedures); and uncertainty from findings of unknown clinical significance will lead to unnecessary investigations and anxiety for the patient. All these translate to financial and psychological burden to the patient, as well as financial and other burdens to the healthcare system. Finally as discussed above, the risks from radiation are real and significant.

Theoretically, the perceived benefits are earlier detection leading to earlier intervention, reduction of morbidity and mortality for the patient, and lower overall health costs and burden to the healthcare infrastructure. However, these benefits have not been proven yet. Therefore, the risks outweigh the benefits in whole body screening CT at this time.

Every medical procedure must have indications and justification for its use. Consideration must be given to the available resources versus clinically useful information that alters management inclusive of financial and safety issues. This is even more imperative with medical procedures requiring the administration of ionising radiation.

Justification, optimisation and dose limitation remain the main tenets for radiology practitioners. In clinical scenarios where benefit exceeds risks and further management of the patient is dependent on information gleaned from the CT scan, the examination is deemed justified. The radiologist and the radiographer/radiologic technologist operating the CT scanner must optimise the examination to get the most information by using scan parameters that do not use excessive radiation (dose limitation). CT scans should be performed keeping in mind the principle of ALARA (As Low As Reasonably Achievable).

In Malaysia, all CT scans must be conducted by a trained medical practitioner who is a qualified radiologist with a valid practicing licence from the Malaysian Medical Council (MMC) and is preferably listed in the National Specialist Register (currently a voluntary registration process). The equipment must be operated by trained and qualified radiographers. The facility providing the CT service must have in place radiation protection and quality assurance programmes.

## SPECIAL CIRCUMSTANCES FOR TARGETED SCREENING CT PROCEDURES

This position statement would be incomplete without also discussing some of the results of studies done for screening CT confined to specific areas of the body, as well as for diseases which are deemed common and for which earlier intervention may impact positively on morbidity and mortality.

### A. CT Heart for Calcium Score

Calcium scoring in cardiac CT is deemed appropriate for patients at medium risk for coronary artery disease, as the information from the calcium score may render the patient as high risk, requiring more intensive risk modification [11]. More recently, calcium scoring may prove to be independently predictive of cardiovascular risk and adds incremental prognostic information to the conventional risk factor scoring methods [11].

### B. CT colonography (Other names are virtual colonoscopy and virtual colonography)

This is a CT examination of the colon, and typically involves distending the colon with carbon dioxide or air, and then performing a CT scan of the abdomen and pelvis, usually in both prone and supine positions. The data is reconstructed and can provide axial images, 3-D, MPR images as well as colon fly-through video images. The radiation dose in CT colonography ranges from 1.8 mSv to 15 mSv with an average of 8 mSv. The radiation dose in barium enema (barium study using x-ray fluoroscopy) is typically 7 mSv.

Recently, CT colonography was recognised as a screening tool in the Joint Guideline of the American Cancer Society, the US Multi-Society Task Force on Colorectal Cancer and the American College of Radiology on Screening and Surveillance for the Early Detection of Colorectal Cancer and Adenomatous Polyps, 2008 [12, 13]. Screening average-risk individuals over 50 years old may reduce mortality from colorectal cancer [13]. Screening should proceed in a proper programme that begins with risk stratification, and based on the findings, the results from the initial test should be followed through appropriately. For this screening examination to be effective, the patient must adhere to the programme and undergo good quality tests. CT colonography must be used judiciously and existing recommendations for screening in low, medium and high risk individuals should be factored into the decision to proceed with screening CT colonography.

### C. Screening lung CT is still controversial [14, 15, 16, 17, 18].

Although CT is probably best at detecting early lung cancer, the evidence to suggest that treatment / intervention at this stage reduces mortality or improves life span is not conclusive [19]. In addition, lung CT detects many small benign nodules, and the cost-safety-benefit analysis is not straightforward, as lung biopsy carries significant risks. There are ongoing trials in screening lung CT in the at-risk population, including the National Lung Screening Trial in the USA [20]. However, even trials are surrounded by controversy. The International Early Lung Cancer Action Programme was recently reported to have received some funding from a tobacco company [21]. The latter adds to the other questions on lead time bias, study length and over diagnosis bias.

## CONCLUSION

After an extensive review of the published literature, the CoR at this time does not recommend whole body screening CT scans in healthy asymptomatic individuals, as the risks (safety, psychological, cost) outweigh the potential benefits (reduced mortality, burden and costs to healthcare systems). The appropriateness of screening CT examinations may change with new evidence, improvements or changes in CT technology, disease pattern, type, treatment, and various other factors. [12-28].
